# Techniques for Mitigating the Effects of Smoke Taint While Maintaining Quality in Wine Production: A Review

**DOI:** 10.3390/molecules26061672

**Published:** 2021-03-17

**Authors:** Ysadora A. Mirabelli-Montan, Matteo Marangon, Antonio Graça, Christine M. Mayr Marangon, Kerry L. Wilkinson

**Affiliations:** 1 Department of Agronomy, Food, Natural Resources, Animals and Environment (DAFNAE), University of Padova, Viale dell’Università 16, 35020 Padova, Italy; ysadoraashton.mirabellimontan@studenti.unipd.it (Y.A.M.-M.); christine.marangon@unipd.it (C.M.M.M.); 2 Sogrape Vinhos S.A., Aldeia Nova, 4430-809 Avintes, Portugal; antonio.graca@sogrape.pt; 3 Department of Wine Science and Waite Research Institute, The University of Adelaide, PMB 1, Glen Osmond, SA 5064, Australia; kerry.wilkinson@adelaide.edu.au; 4 The Australian Research Council Training Centre for Innovative Wine Production, PMB 1, Glen Osmond, SA 5064, Australia

**Keywords:** amelioration, climate change, grapes, smoke taint, volatile phenols, wildfires, wine

## Abstract

Smoke taint has become a prominent issue for the global wine industry as climate change continues to impact the length and extremity of fire seasons around the world. Although the issue has prompted a surge in research on the subject in recent years, no singular solution has yet been identified that is capable of maintaining the quality of wine made from smoke-affected grapes. In this review, we summarize the main research on smoke taint, the key discoveries, as well as the prevailing uncertainties. We also examine methods for mitigating smoke taint in the vineyard, in the winery, and post production. We assess the effectiveness of remediation methods (proposed and actual) based on available research. Our findings are in agreement with previous studies, suggesting that the most viable remedies for smoke taint are still the commercially available activated carbon fining and reverse osmosis treatments, but that the quality of the final treated wines is fundamentally dependent on the initial severity of the taint. In this review, suggestions for future studies are introduced for improving our understanding of methods that have thus far only been preliminarily investigated. We select regions that have already been subjected to severe wildfires, and therefore subjected to smoke taint (particularly Australia and California) as a case study to inform other wine-producing countries that will likely be impacted in the future and suggest specific data collection and policy implementation actions that should be taken, even in countries that have not yet been impacted by smoke taint. Ultimately, we streamline the available information on the topic of smoke taint, apply it to a global perspective that considers the various stakeholders involved, and provide a launching point for further research on the topic.

## 1. Introduction

Wildfires represent a significant climate issue around the world, with implications for land use and public safety. The incidence and severity of wildfires in fire-prone areas have not only increased in recent years, but fires have begun to affect new regions [1]. Each year, nearly 350 million hectares of land are burned across the globe [2]. According to the National Oceanic and Atmospheric Administration’s 2019 annual Global Climate Report, the nine warmest years on record (i.e., since 1880) have occurred in the last 15 years, with 2016 having the highest global surface temperature to date, being 0.99 °C above average [3]. In the United States, around 7.5 million acres (~3 million hectares) of land have been impacted by wildfires annually since 2011, with 2020 being the worst affected year, during which 10.3 million acres (~4 million hectares) burned; 40% of which was in the state of California [4]. In Europe, the Mediterranean region (i.e., Portugal, Greece, Italy, Spain, and southern France) is particularly affected by fires. More than 95% of these fires are caused by human activity and many can be attributed to poorly executed use of traditional practices involving intentional burning of shrubs/straw. Approximately 85% of the half a million hectares of land burned in Europe annually are contained within the Mediterranean region. The majority of fires that occur in the Mediterranean occur between June and October [5], such that the timing of fires poses a serious threat to grape production in those areas. Much of Australia’s landscape has the natural propensity to burn, placing it at a significant risk of wildfire danger. As stated by the Bureau of Meteorology, 2019 was Australia’s hottest and driest year on record, with average national temperatures surging past the previous record high of 40.3 °C in January 2013, reaching 41.9 °C in December 2019 [6]. During the 2019–2020 fires that occurred in Australia, more than 17 million hectares of land burned, i.e., more than 8 times the area that burned during the historic ”Black Friday” fires in Victoria, Australia in 1939 [7]. With the frequency of heat waves and droughts predicted to increase, the likelihood of wildfires occurring around the world will also increase [8].

It is widely recognized that the exacerbation of fire incidents can be attributed to climate change, compounded by many factors, including hot, dry, and windy weather conditions; decreased rainfall leading to extended periods of drought; and increased fuel loads which depend on land and fire management practices [9,10,11,12,13]. Some of the most prominent wine regions in the world, including those in Australia, Canada, Chile, South Africa, and the United States are experiencing climate pressures, and wildfires have caused serious problems for the wine industry, including crop loss and vineyard damage due to burning and/or smoke exposure [9,14,15,16,17]. As climate change continues, the occurrence of wildfires is expected to increase in frequency and severity, and to affect winemaking regions that have not yet been severely impacted [18,19]. Parts of southern Europe (in particular, Spain, Italy, and Portugal) have experienced wildfires (especially in 2017–2018) and these regions are predicted to experience more frequent wildfires, with worsening severity in coming years [10,20,21]. As the incidence of wildfires increases, and periods of drought and fire extend (both in duration and geographical expanse), so too will the fire-related pressures on agricultural production. Furthermore, there are stakeholders with competing interests with regards to fire and land management practices, which can cause secondary problems to arise. For example, unintentional smoke and/or fire damage from prescribed burns, which, depending on their timing, can have detrimental effects on agricultural crops, including grapes for wine production [19,22,23].

Though wildfires can cause many problems for winemakers (beyond the obvious concerns for public safety), such as property loss, crop loss, and smoke taint, in this article, we focus specifically on the issue of smoke taint. When grapevines are exposed to smoke, their leaves and fruit can adsorb volatile smoke compounds (for example, volatile phenols such as guaiacol, 4-methylguaiacol, o-, m- and p-cresol, and syringol), which can initially be detected in free (aglycone) forms but are rapidly converted to glycoconjugate forms due to glycosylation [11,24,25,26,27,28,29,30]. These glycoconjugates can be broken down and the volatile phenols released during the fermentation process, causing undesirable sensory characteristics (i.e., smoky and ashy attributes) in the resultant wines [11,31]. Although the exact pathway by which smoke volatiles are taken up has not yet been definitively proven, an isotope tracing experiment suggested translocation of these compounds (in free or glycoconjugate forms) between the various parts of the grapevine is minimal [25]. However, elevated volatile phenols were detected in wines made from grapes that were exposed to just 30 min of smoke exposure during the growing season [32]. Thus, it is likely that there is a direct and immediate pathway of adsorption into both grapevine leaves and fruit.

There is still some confusion as to which smoke-derived volatile compounds are responsible for the taint perceived in wines made from smoke-affected grapes, and the number of compounds that contribute to smoke taint might be vast and complex [33,34]. Early studies have measured guaiacol and 4-methylguaiacol as markers of smoke taint, because these compounds were routinely identified in wines aged in oak barrels, as metabolites of the thermal degradation of lignin that occurs during barrel toasting [35], and so analytical methods were readily available for their quantification [36]. These compounds impart smoke aromas and flavors to oak-aged wines [37,38,39], with their contribution to wine generally considered to be positive, i.e., without any suggestion of smoke taint. As smoke taint research has progressed, the range of volatile phenols that were measured as smoke taint markers evolved to include cresols, phenol, and syringols, in addition to guaiacol and 4-methylguaiacol, and analytical methods were developed to measure both free and bound (glycosylated) volatile phenols [27,33,40]. Several studies have attempted to establish the sensory contributions of smoke-derived volatile phenols [41,42,43]; while the volatile phenol glycoconjugates that remain in wine after fermentation [33,44] are thought to contribute to the ashy aftertaste perceived in some smoke-tainted wines, due to in-mouth hydrolysis [45]. Nevertheless, it is reasonable to expect that additional smoke taint marker compounds might be identified in the future.

A number of methods have been evaluated, both preventative viticultural measures and ameliorative winemaking techniques, for mitigating and/or remediating the negative effects of grapevine smoke exposure. However, there is currently no single method that universally solves the problem of smoke taint. The timing and duration of smoke exposure during each fire incident [15,32], as well as grape variety [44] and desired style of wine [46] have all been shown to influence the extent of smoke taint in the resultant wine, thus, the exact method of remediation must be carefully examined on a case-by-case basis.

The aim of this paper was to analyze the available methods for minimizing the negative effects of smoke-derived taint in grapes and wine, while maintaining the quality of the final product. This was achieved by reviewing the literature currently available on smoke taint as follows: Firstly, by outlining the key discoveries made over the past fifteen years that contributed to our current understanding of smoke taint, and then by summarizing the efficacy of the methods for prevention and remediation of smoke taint. This investigation comprised a global perspective, with the intention of using the more severely affected winemaking regions (namely, those in California and Australia), to provide insights for other winemaking regions (particularly regions in southern Europe) that will likely become more impacted as the effects of climate change intensify over time.

## 2. Key Discoveries in Smoke Taint Research

Smoke taint is still a relatively young field of research, having only started systematically ~18 years ago. The first peer-reviewed paper on smoke taint was published in the scientific literature, in 2007, by Kennison et al. [47]. The study described an experiment conducted in Western Australia in which bunches of Verdelho grapes were exposed to smoke postharvest, fermented, and the resultant wines were evaluated by chemical and sensory analysis [47]. Importantly, this study demonstrated (for the first time) that exposure to smoke could negatively impact wine composition and quality, leading to a perceivable taint, characterized by objectionable smoky, dirty and burnt aromas and flavors. Several volatile compounds usually associated with oak maturation, i.e., guaiacol, 4-methylguaiacol, 4-ethylguaiacol, 4-ethylphenol, eugenol, and furfural [36] were detected in wines made from the smoke-affected grapes, but not in the corresponding control wines. As such, their presence was directly attributed to the application of smoke to fruit. Among the compounds measured, guaiacol and 4-methylguaiacol were the most abundant. However, the authors suggested that, while these compounds were useful markers of smoke taint, they were unlikely to be solely responsible for the sensory perception of smoke taint and additional smoke-derived volatile compounds would likely be identified in subsequent research [47].

In the following year, Kennison et al. published an experiment involving the application of smoke to Merlot grapevines grown in a vineyard in Capel, Western Australia [11]. Chemical analysis was performed on samples collected during the fermentation of control and smoke-affected grape must, and the concentration of several smoke-derived volatile phenols (including guaiacol, 4-methlyguaiacol, 4-ethylguaiacol, and 4-ethylphenol) were found to increase progressively throughout primary and secondary fermentation, and importantly, even after wines were pressed from the skins. This confirmed anecdotal evidence from winemakers that smoke characters appeared/intensified during the winemaking process and provided the first evidence for accumulation of smoke-derived volatile phenols in precursor forms. Enzyme and acid hydrolysis experiments confirmed additional quantities of volatile phenols could be released from smoke-affected juice, but not control juice. The authors concluded that the evolution of volatile phenols following treatment of smoke-affected juice with β-glucosidase suggested the precursors were glycoconjugates. Subsequent research (by Australian and Canadian researchers) confirmed the presence of the β-D-glucopyranoside of guaiacol in juice from smoke-affected grapes [26], and then glycoconjugates (glucoside, glucose-glucosides, pentose-glucosides, and rutinosides) of guaiacols, cresols, and syringols [27,40], which led to the development of analytical methods for the quantification of volatile phenol glycoconjugates [25,40,48,49].

In 2009, Kennison et al. performed a series of field trials (again in Capel, Western Australia) to investigate the effects of the timing and duration of grapevine smoke exposure on the composition and sensory properties of wine [32]; single smoke treatments were applied to Merlot grapevines at eight time points between veraison and harvest, while repeated smoke treatments were also applied to vines at each of the same eight time points. This study demonstrated the following: (i) Repeated smoke exposure had a cumulative effect on the concentration of volatile phenols and the sensory perception of smoke taint in wine and (ii) grapevines appeared to be more susceptible to smoke when exposure occurred seven days post veraison, albeit, smoke attributes were perceived to varying degrees in all of the wines made with fruit from smoke-exposed grapevines [32]. Similar findings were obtained when field trials were repeated in subsequent seasons [15], with smoke treatments applied from E–L stage 12 (when shoots were ~10 cm) to E–L stage 38 (harvest). However, this has not been investigated in other cultivars or in other grape growing regions. The latter study also investigated the potential for smoke taint to be carried over from one growing season to the next, but smoke-derived volatile phenols were not detected in wines made with fruit from grapevines that were exposed to repeated smoke treatments in the previous growing season [15]. These discoveries were important because they confirmed that the duration and timing of smoke exposure impacts the severity of smoke taint, implying that the occurrence of a fire event near a vineyard does not necessarily mean the resultant wine will exhibit a perceivable taint. Strategies for monitoring grapevine smoke exposure in the vineyard, as well as screening of grape samples prior to vinification, therefore, have been evaluated, enabling winemakers to better predict the risk of smoke taint occurring in finished wine [29,50].

In 2010, Hayasaka et al. published findings from a series of studies (undertaken in Australia) that investigated the conjugation of smoke-derived volatile phenols in grapes [25,26,27]. The first of these studies was a progression of the earlier work by Kennison et al. [11] and demonstrated the existence of guaiacol in precursor forms in smoke-affected grapes [26]. The β-d-glucopyranoside of guaiacol was synthesized and used as a reference standard to confirm its presence in juice of fruit from smoke-affected grapevines, and absence in the corresponding control juice. This confirmed that guaiacol was taken up by grapes and subsequently glycosylated, following grapevine exposure to smoke. Acid and enzymatic hydrolyses were also conducted to investigate the breakdown of the guaiacol β-d-glucopyranoside. Both were capable of hydrolyzing the β-d-glucopyranoside to release guaiacol, with more complete hydrolysis observed during enzymatic hydrolysis, confirming the role of fermentation in breaking down these glycoconjugates. However, acid hydrolysis of juice from smoke-affected grapes released more guaiacol than enzymatic hydrolysis, indicating the likelihood that other guaiacol precursors that were less susceptible to hydrolysis by β-glucosidase were present in the grapes [26].

In a separate study, Hayasaka et al. further investigated the glycosylation process using an isotope tracing experiment, which identified additional glycoconjugate precursors of guaiacol, in both grapes and leaves [25]. A number of different guaiacol glycoconjugates, in addition to the β-d-glucopyranoside were putatively identified, including glucose-glucosides, pentose-glucosides, and rutinosides; differences in their relative abundances were observed between leaves and berries. The identity of a range of volatile phenol glycosides was confirmed in a subsequent study (conducted in British Columbia, Canada), involving synthesis, and then fragmentation analysis using high-resolution, accurate mass spectrometry [28]. The Hayasaka study also examined the potential for translocation of these compounds, by comparing the compositional consequences of applying an aqueous mixture of d0- and d3-guaiacol directly to grapevine leaves and berries, relative to the background levels observed in a control vine with no guaiacol application [28]. Very little translocation of guaiacol was found, either leaf to leaf, bunch to bunch, or between leaves and bunches, which suggested that guaiacol was more likely adsorbed directly by the grapes and leaves. Interestingly, the control juice also contained low levels of guaiacol glycoconjugates, despite no guaiacol being applied to the vines or berries, demonstrating the natural occurrence of guaiacol in some cultivars. Furthermore, the guaiacol glycoconjugates were detected in the grape skins, and were also present in the pulp [25].

The third study, published by Hayasaka et al., in 2010, explored the occurrence of volatile phenol glycosides other than guaiacol in grapes exposed to smoke from a prescribed burn in the Adelaide Hills (South Australia), as well as the release of free volatile phenols from their glycosylated precursors during winemaking and storage. This study found that “volatile phenols from bushfire smoke, including phenol, cresols, methylguaiacol, syringol, and methylsyringol, can be metabolized to glycoconjugate forms within grapes in a similar fashion to that shown previously for guaiacol.” The study also demonstrated that these volatile phenols could be released into wine at significant concentrations (i.e., over 100 µg/L) when phenolic glycosides were present in juice at concentrations of 20 mg/L; strong acid hydrolysis conditions released ten-fold higher volatile phenol concentrations (~1000 µg/L), which the authors concluded might reflect the potential for hydrolysis to occur naturally during wine storage/aging [27].

In 2011, Singh et al. investigated the presence of guaiacol and 4-methylguaiacol in bound forms in bottled wines produced from grapes sourced from vineyards affected by bushfires in the King Valley region (Victoria), and the potential for bound volatile phenols to serve as an “aroma reserve” for smoke taint [51]. This study showed that bound compounds could possibly hydrolyze during bottle aging, by way of acid hydrolysis, to release volatile phenols, with the potential to lead to the increased perception of smoke taint. Additionally, this study validated a GC-MS method to monitor guaiacol and 4-methylguaiacol (both free and bound) in grapes and wine, after release by acid hydrolysis [51]. However, in 2017, a more detailed study investigating changes in chemical and sensorial properties of smoke-tainted wines after six years of bottle aging was published [52]. White and red wines (multiple varieties) were made from fruit harvested from grapevines exposed to smoke using purpose-built smoke tents. Chemical analysis showed no significant changes in total guaiacol glycoconjugate concentrations post bottle aging, and similar changes in volatile phenol concentrations between control and smoke-tainted wines. Some changes were observed in the perceived intensity of smoke-related sensory characteristics in wines, which the authors attributed more to the decrease in varietal fruity expression than the release of smoke-derived volatile phenols from their glycoconjugate precursors forms. This study revealed that the glycoconjugates of smoke-derived volatile phenols are actually relatively stable and require significant heat and/or strong acid to hydrolyze [52]. However, while acid hydrolysis during storage does not greatly impact the release of smoke-derived volatile compounds, another study by Mayr et al. discovered that enzymatic hydrolysis can be activated by saliva inside the mouth, releasing undesirable smoke aromas and flavors, which can be perceived (to varying degrees) by the person drinking wine containing glycoconjugates of smoke-derived volatile phenols [45]. This suggests that consumers might still perceive smoke taint in wines if the glycoconjugate forms are not fully removed, as a result of in-mouth enzymatic breakdown [45]. This could be an important consideration when deciding whether or not to release wine produced from smoke-affected grapes into the market.

In 2012, Kelly et al. published an experiment that investigated differences in volatile profiles of wines produced from grapes from a vineyard in Margaret River (Western Australia) that were exposed to smoke derived from the combustion of fuels (jarrah, karri, marri, radiata pine, and wild oats) comprised of different lignin compositions [34]. Kelly hypothesized that because volatile phenols were derived from the pyrolysis of lignin present in the fuel source, the volatile phenol profiles of wines should differ depending on the composition of the fuel being burned [34]. On the basis of the results of this study, the authors concluded that there were likely many more compounds contributing to smoke taint than had previously been identified in earlier studies. The authors also suggested that p-hydroxyphenols and syringols might be responsible for the sensory defects observed in smoke-tainted wines. This review highlights a particular challenge associated with addressing the remaining knowledge gaps on this subject, i.e., that the unpredictable nature of wildfires means it is difficult to predict the fuel(s) that will be burned, and thus, the composition of smoke that might drift into vineyards during a fire event. Therefore, it is difficult to ascertain which smoke-derived volatile compounds might be most responsible for contributing taint to wine made from smoke-affected grapes. Smoke-derived volatile phenols (and their glycoconjugates), nevertheless, provide useful markers of smoke taint in grapes and wine. However, wine producers and wine researchers alike need to be mindful of the occurrence of some volatile phenols as either natural constituents of certain grape cultivars, Shiraz/Syrah in particular [53,54], or the oak used to make barrels [36], which can confound the detection and quantification of smoke taint in wine.

Recently, Caffrey et al. confirmed the complexity associated with smoke taint in a study that investigated the diversity of volatile phenol glycosides present in grapes (from the Napa Valley, California) that were exposed to wildfire smoke for an extended period of time [33]. The study identified thirty-one volatile phenol glycosides (including a number of trisaccharides) in grapes and fermenting must that were tentatively attributed to smoke taint. The existence of a vast array of volatile phenol glycosides that may contribute to the undesirable characteristics of wines made from smoke-affected grapes indicates the need for a better understanding of the sensory contribution of the various compounds derived from smoke exposure.

Despite the remaining gaps in our understanding of the impacts of grapevine smoke exposure, the advancements made in this field of research in less than two decades are remarkable, especially considering these issues have mostly been investigated in Australia, the USA, Canada, and South Africa (regions that have been regularly exposed to wildfire events in recent years). Collectively, the discoveries made to date lay the groundwork for future research to be undertaken globally, which will hopefully yield a universal remedy for smoke taint.

## 3. Methods to Minimize the Negative Impacts of Smoke Taint

**Figure 1:**
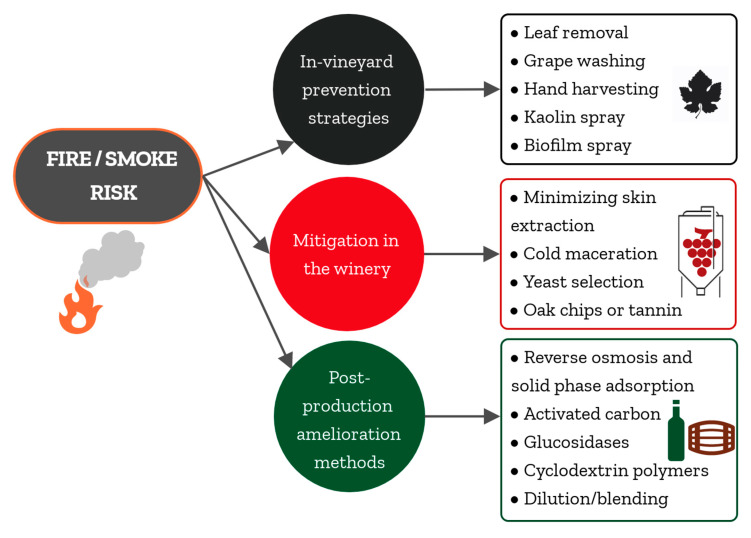
Summary of the different strategies used to reduce the effects of smoke on wine composition and sensory quality.

In order to minimize the negative impacts of wildfire events on the wine industry, researchers have evaluated different strategies for mitigating the effects of smoke on wine composition and sensory quality (Figure 1).

Amelioration strategies have focused on the following: (i) mitigating the uptake of smoke volatile compounds during grapevine exposure to smoke in the vineyard, (ii) minimizing the extraction of smoke taint compounds into juice or must by adopting different grape processing techniques in the winery, or (iii) removing the compounds responsible for smoke taint from finished wine after fermentation. In the following sections, these methods, and their benefits, drawbacks, and limitations, are presented.

### 3.1. Vineyard-Based Prevention Strategies

A number of preventative strategies have been evaluated to establish whether or not the issue of smoke taint can be addressed in the vineyard. Research has considered vineyard practices that can be conducted prior to smoke exposure, as well as at the time of harvest, with the aim of minimizing the effects of smoke exposure before harvesting and processing the fruit. Preventative methods that have been explored included the following: washing grapevines/grapes, partial leaf removal, the application of agricultural sprays, and different harvesting techniques (i.e., hand-harvesting vs. machine harvest) (Table 1).

**Table 1:** Summary of the methods evaluated for prevention of smoke taint in the vineyard.

Method	Key Findings	Variety and Location	Effectiveness
**Washing grapes during/after smoke exposure**	Washing vines or grapes with water, aqueous ethanol, or milk after smoke exposure did not affect the guaiacol content of grapes or juice. Misting grapes during smoke exposure partially mitigated the uptake of volatile phenols by grapes but did not influence the perception of smoke taint in wine [29,40,55].	Cabernet Sauvignon, Cabernet Franc, Chardonnay (Australia)	None–Low
**Leaf removal prior to or after smoke exposure**	Where grapevines were partially defoliated before smoke exposure, wines exhibited more intense smoke characteristics. Where grapevines were partially defoliated after smoke exposure, wines exhibited more intense fruit characteristics which helped mask smoke attributes. However, this did not eliminate the taint, and should be paired with other methods [56].	Chardonnay (Australia)	None
**Hand-harvesting fruit**	Preventing leaves, which can adsorb smoke-derived volatile compounds from entering the must avoids extraction of additional taint compounds. However, this will not prevent extraction of taint compounds already present in grapes and should therefore be paired with other methods [57,58,59].	Pinot Noir, Merlot (Canada, Australia)	Low
**Application of kaolin to vines**	There was no conclusive evidence that applying kaolin to grapevine fruit and foliage prior to smoke exposure provided protection; results varied depending on grape variety and spray coverage [30,60].	Sauvignon Blanc, Chardonnay, Merlot, Pinot Noir (Australia)	More information needed
**Application of biofilm to vines**	Preliminary results were promising and suggested that applying biofilm to grapevine fruit and foliage prior to smoke exposure provides protection, but more information is needed regarding the efficacy of the spray and the feasibility of application before a fire incident [57].	Pinot Noir (Canada)	More information needed

Some of the earliest attempts to mitigate the effects of grapevine exposure to smoke involved washing grapevines or fruit with water, 5% aqueous ethanol, or milk [28,55], but these strategies did not significantly influence the guaiacol concentration of grapes or juice. In a more recent study, Szeto et al. evaluated in-canopy misting as a strategy to mitigate the uptake of smoke-derived volatile compounds [29]. A sprinkler system mounted in the grapevine canopy facilitated washing of grapevines during exposure to smoke, in an attempt to mimic the atmospheric cleansing of aerosols that occurs when it rains. However, despite some differences in the volatile phenol glycoconjugate profiles of grapes, the misting treatment did not affect the concentration of volatile phenols observed in wines or the sensory perception of smoke taint. The authors concluded this might reflect the speed with which smoke-derived volatile phenols diffuse into grape berries.

A study published by Ristic et al., in 2013, compared the effects of partial defoliation of vines performed before and after smoke exposure [56]. The study showed that where defoliation occurred prior to smoke exposure, wines exhibited more intense smoke sensory attributes, relative to control wines (i.e., wines corresponding to grapevines that were not exposed to smoke, with or without partial defoliation), as well as increased levels of smoke-derived volatile phenols and glycoconjugates. Where defoliation occurred after smoke exposure, wines showed less intense smoke taint due to increased fruit characteristics (which the authors hypothesized masked some of the smoke characters). The exact causes of these differences were not conclusively identified in this study, but were hypothesized to reflect a physiological response, possibly due to differences in berry temperatures caused by sun exposure as the result of defoliation leading to enhanced metabolic activity [56]. Nevertheless, this study provided some evidence that partial defoliation following a fire event could impact the extent to which grapes might be tainted. However, whether this approach is practical, especially where there are ongoing safety concerns during a prolonged fire event, is questionable.

A number of studies have recommended hand-harvesting grapes rather than machine harvesting, to avoid breaking berries prematurely and facilitating the extraction of smoke taint compounds from grape skins [57,58,59]. This also prevents the incorporation of leaves into fermentations, and therefore the extraction of additional smoke taint compounds [57,58,59]. In general, this is considered to be good winemaking practice, but in the case of smoke taint, this approach can help to limit the concentration of smoke taint compounds present in fermenting juice or must. However, this does not address the smoke taint compounds that are already present in the grapes, and therefore this approach would need to be paired with other amelioration techniques (presented in Section 3.2 and Section 3.3), either during or after winemaking.

Other key vineyard-based preventative strategies, which have been studied to date, have involved the application of agricultural sprays to grapevine foliage, with varying degrees of success. In 2019, van der Hulst et al. evaluated the application of kaolin (a clay-based material) to grapevine foliage/fruit [30]. The study yielded mixed results between cultivars, with a promising outcome for Merlot grapes, but no real effect in Chardonnay or Sauvignon Blanc. This disparity was thought to be due to varying levels of spray coverage achieved between cultivars [30]. As a consequence, the efficacy of kaolin was inconclusive and further research is needed, including determining the sensory impact of kaolin treatment of vines (albeit kaolin is already used in grape production as a sun protectant). A more recent study looked at the cuticular wax of grapes and their apparent ability to insulate the berries as well as facilitate the passage of various compounds [57]. Field trials evaluated three different treatments to grapevines, i.e., two different fungicidal oils and a ”biofilm” described as “an artificial phospholipid cuticle designed to prevent fruit-cracking in soft-fleshed fruits.” On the one hand, neither of the fungicidal oils prevented the uptake of smoke-derived volatile phenols, in fact, one of the oils used (a tea tree oil) seemed to exacerbate the effects of smoke exposure. The biofilm, on the other hand, significantly decreased the uptake of smoke-derived compounds, showing promise as a preventative measure to mitigate the extent of smoke taint. The authors, however, acknowledged that further research would be required before the biofilm could be considered to be an adequate prevention method [57]. Furthermore, the practicality of applying agricultural sprays prior to a fire event is again questionable, due to safety concerns.

Preliminary research has also been conducted into postharvest ozone fumigation of grapes as a method of reducing guaiacol and 4-methylguaiacol concentrations in wine, thereby minimizing the sensorial impact of smoke taint [61]. However, this method needs to be investigated further to determine how effectively it mitigates smoke taint, and to what extent ozone influences wine color and desirable aromas and flavors.

### 3.2. Grape Processing Methods

A number of fruit processing methods have been considered to be strategies to minimize the negative effects of smoke exposure in the resultant wine, including the duration of skin contact, the maceration/fermentation temperature, the strain of yeast selected for fermentation, as well as the addition of oak chips and tannins (Table 2).

**Table 2:** Summary of the methods evaluated for mitigation of smoke taint in the winery.

Method	Key Findings	Variety & Location	Effectiveness
**Minimizing** **extraction from skins**	Shorter maceration times, whole bunch pressing, and separating press fractions can help to reduce the extraction of smoke taint compounds from grape skins but limits the wine styles that can be made [46,59].	Grenache (Australia)	Low–moderate
**Cold maceration**	Cold maceration can help to reduce the extraction of smoke taint compounds but limits the wine styles that can be made. Does not eliminate the taint, just reduces the perceived intensity in wine [46,59].	Grenache (Australia)	Low
**Yeast selection**	Different winemaking yeast can enhance desirable organoleptic characteristics, thereby masking smoke attributes. Does not eliminate the taint but can reduce the perceived intensity in wine [46].	Grenache (Australia)	Low
**Addition of oak chips or tannins**	Addition of oak chips or tannin can help to mask smoke taint but does not remove smoke taint compounds and are only effective for mildly smoke-affected grapes, otherwise must be paired with other methods that can remove smoke taint compounds [46,59].	Shiraz (Australia)	Low

Ristic et al. investigated different winemaking techniques with varying results [46]. Implementation of a cold maceration method, with limited skin contact (compared with traditional fermentation on skins) had a significant impact on the levels of guaiacol and 4-methylguaiacol that were detected in the final rosé-style wine. This strategy favors some wine styles over others, in particular, rosé and white wine production, but the risk of smoke taint increases with red wine production, which requires a longer duration of skin-contact for extraction of anthocyanins and other organoleptically desirable phenolic compounds responsible for red wine color and mouthfeel properties. The Ristic study also showed that the addition of particular tannins and oak additives could distract from the perception of smoke taint, “albeit through increased wine complexity, rather than the reduction in concentration of smoke-derived volatile phenols.” Furthermore, this study evaluated the extent to which different yeast strains influenced the level of smoke taint in finished wine. Compositional and sensory differences were observed among wines made with different yeast strains; in some cases, smoke attributes were enhanced and, in other cases, they diminished, but none of the yeast strains studied were capable of eliminating the perception of smoke taint [46].

A more comprehensive overview of winemaking techniques for minimizing the incidence of smoke taint in wines has been published by the Australian Wine Research Institute [59]. This includes recommendations that aim to reduce the extraction of smoke taint compounds from the skins through shorter maceration times, the use of whole bunch pressing, and separation of press fractions. These suggestions, especially when applied in combination with techniques that remove smoke taint compounds from wine (as discussed below) contribute to limiting the intensity of smoke taint, but their application inherently limits the types of wine that can be produced.

### 3.3. Post-Production Methods

A number of post-production amelioration techniques involving fining and/or filtration have previously been, and continue to be, studied, due to their promising results. Several of these post-production techniques are currently used commercially to treat smoke-tainted wines (Table 3).

**Table 3:** Summary of the methods evaluated for post-production amelioration of smoke taint in wine.

Method	Key Findings	Variety & Location	Effectiveness
**Reverse osmosis and solid phase adsorption**	This method reduced the concentration of smoke-derived volatile phenols in wine, but volatile phenol glycoconjugates were not removed and might still impart perceivable taint characters. This approach may not salvage severely smoke-tainted wine [62].	Pinot Noir (Australia)	Moderate
**Addition of activated carbon**	Activated carbon can remove smoke-derived volatile phenols from wine, with some preliminary evidence suggesting that certain activated carbons might also remove volatile phenol glycoconjugates. This appears effective for treating mildly smoke-tainted wines, but cannot remedy severely tainted wines, and without removal of glycoconjugates, taint might still be perceived. Some activated carbons also strip wine color and/or desirable volatile compounds (aroma and flavors) from wine [58,63,64].	Pinot Noir, Cabernet Sauvignon, Merlot, Chardonnay (Australia)	Moderate
**Addition of glucosidases**	Preliminary studies involving addition of glucosidase enzymes to hydrolyze volatile phenol glycoconjugates, enabling the resulting volatile phenols to be more easily removed via other methods of amelioration (e.g., reverse osmosis or activated carbon treatments), offered little evidence of success. More research is needed to evaluate the efficacy of other glucosidases to achieve this purpose [58,65].	Pinot Noir, Cabernet Sauvignon, Merlot, Shiraz, Chardonnay (Australia)	None
**Addition of cyclodextrin polymers**	Two cyclodextrin polymers were evaluated and found to be capable of adsorbing from 45 to 77% of four volatile phenols studied. Additionally, CD polymers can be regenerated. The efficacy of the method for removal of volatile phenol glycosides still needs to be assessed [66].	Cabernet Sauvignon (Australia)	Moderate
**Dilution** **/Blending**	Blending or dilution of smoke-tainted wine with a base (unaffected) wine can diminish the intensity of smoke taint to levels that are comparable to the base wine alone. However, the level of dilution required depends on the initial concentration of smoke taint compounds present in the wine [47,67].	Verdelho, Pinot Noir (Australia)	Moderate

One of the earliest strategies evaluated as a method for remediation of smoke-tainted wines involved reverse osmosis and solid phase adsorption. The process fractionates wine (nominally on the basis of molecular mass), then selectively treats the permeate fraction (comprising the lower molecular weight smoke taint compounds), before the treated permeate is blended with the retentate fraction to restore the wine. The method has been shown to effectively remove smoke-derived volatile phenols from tainted wines [62], which improved the sensory properties of the wine. The authors monitored changes in the volatile phenol concentration of treated wine over time and initially interpreted a temporal increase in volatile phenols as the return of smoke taint over time, due to acid hydrolysis of glycoconjugates, which did not permeate the reverse osmosis membrane [62]. The stability of glycoconjugates during bottle aging [52] suggests changes in volatile phenol levels may not reflect the return of smoke taint and it may have occurred irrespective of grapevine smoke exposure (i.e., naturally). In a subsequent study, Fudge et al. evaluated the removal of volatile phenols through addition of different commercial fining agents [63]. This study identified two fining agents that gave promising results, i.e., an activated carbon and a synthetic mineral, with the activated carbon offering the greatest removal of smoke-derived volatile phenols [63]. However, a key issue with these adsorbents was their apparent specificity for removal of smoke-derived volatile phenols, but not volatile phenol glycoconjugates (at least for the specific fining agents that were evaluated). Thus, glycoconjugates that remained in treated wine could potentially contribute perceivable smoke taint characteristics via in-mouth hydrolysis [45]. Additionally, it should be noted that since activated carbon is a non-specific fining agent, it is also capable of removing other desirable wine constituents, alongside those responsible for smoke taint [58,62,68].

Research into mitigation and remediation of smoke taint is ongoing, and research groups around the world are continuing to evaluate strategies for removing both volatile phenols and their glycoconjugates. The two key approaches taken have relied on the use of (i) adsorbents (e.g., activated carbons) that selectively target the removal of volatile phenols and volatile phenol glycoconjugates (from either juice or wine) and (ii) winemaking yeast, bacteria and/or enzymes that can hydrolyze volatile phenol glycoconjugates to facilitate removal of volatile phenols. Among the different activated carbons that have been tested for their ability to remove glycosides to date, some were found to effectively remove up to 60% of the total glycosides present, but the rate of removal depended on the wine being treated [64]. The ability of various glucosidases (some commercial and some novel) to cleave glycoconjugates has also been evaluated, but with limited success [65]. Krstic et al. suggested that enzyme hydrolysis could be performed in conjunction with secondary treatments, such as reverse osmosis, but that the effectiveness would still depend on the susceptibility of volatile phenol glycoconjugates to enzymatic hydrolysis, as well as the severity of the smoke taint [58].

Recently, the use of crosslinked cyclodextrin (CD) polymers has been investigated for the removal of volatile phenols from wine. Two CD polymers were prepared from β- and γ-CD, with hexamethylene diisocyanate used as a crosslinking agent [66]. The adsorption of four volatile phenols associated with either smoke taint (guaiacol and 4-methylguaiacol) or *Brettanomyces* spoilage (4-ethylguaiacol and 4-ethylphenol) by CD polymers was evaluated, with up to 77% of the volatile phenols being removed in both model and red wine. An advantage of CD polymers is that they can be regenerated and reused. However, to date, the removal of volatile phenol glycoconjugates by CD polymers has not been reported.

The potential for smoke-affected wines to be blended with another wine to dilute or mask the perception of smoke taint has also been evaluated [47,67]. In heavily tainted wines, this is not feasible, because even with a high rate of dilution, smoke taint can still be perceptible [47]. However, this approach was effective in a subsequent study and with sufficient dilution, the sensory profile of the blended wine was not significantly different from the base wine used for blending, alone [67]. Clearly, the suitability of this approach will depend on the severity of smoke taint in the wine and in addition to a blending trial, a cost benefit analysis may need to be performed to determine the financial feasibility of blending. This approach might also be performed in combination with other remediation strategies (e.g., fining with carbon) for removal of some of the smoke taint compounds prior to blending.

## 4. Discussion

Researchers and winemakers alike lament the lack of a single, cure-all solution to the problem of smoke taint. However, given the unpredictable nature of wildfires, the complexity of smoke, and the knowledge gaps remaining regarding the mechanism by which smoke volatiles enter berries and the identity of compounds responsible for organoleptic characteristics associated with smoke-tainted wine, this is perhaps to be expected. Ideally, a single method to remedy smoke taint for all styles of wine will be devised, but for now there are a number of methods that can be implemented, depending on both wine style and the severity of smoke taint (Figure 1 and Table 1, Table 2 and Table 3). Fortunately, there are plenty of avenues for future research. In the final section of this review, we outline the most promising lines of enquiry based on the latest research offerings and suggestions, not just for the countries already impacted by wildfires, but also those countries beginning to experience the strain of climatic changes.

According to the smoke taint research published in the scientific literature to date, it seems that at present, remediation can best be achieved via treatment of wines, post production. Although foliar applications of biofilm and kaolin (Table 1) show potential for mitigating the uptake of smoke taint compounds, the inherent health and safety issues associated with fire events, together with the logistics of implementing applications of agricultural sprays prior to an impending evacuation, make these strategies somewhat risky. The viability of agricultural sprays will depend on the accuracy of fire prediction, and how far in advance sprays can be applied, which, as acknowledged by Favell et al. [57], has yet to be established. Various fruit processing methods have been evaluated, including the use of different yeast strains during fermentation and the addition of oak chips or tannins, but these methods do not remove the taint, rather they enhance varietal character or add complexity to wine, so as to mask the taint. As such, at best, these methods are only applicable for use with mildly to moderately smoke-affected grapes. More severely tainted wines require removal of smoke taint compounds via one or more of the post-production methods of the abovementioned remediation, i.e., treatment with activated carbon, reverse osmosis and solid phase adsorption, blending/dilution, or significantly reduced skin-contact times (but this limits the style of wine that can be produced, which could in turn limit the economic value of the final product).

Because the efficacy of treatment is highly dependent on the initial level of smoke taint, effective methods of analysis (preferably rapid, reliable, and affordable methods) are needed, to enable grape growers and winemakers to establish the severity of smoke exposure after a fire event. Analytical data can be used to inform decisions regarding whether or not grapes should be harvested, and/or what remediation treatments might need to be employed. Currently, the severity of smoke taint is determined analytically by measuring volatile phenols by GC-MS and/or volatile phenol glycoconjugates by LC-MS; bound volatile phenols can also be measured by GC-MS following acid hydrolysis of juice or wine [40,51,53]. Where industry relies on commercial laboratories for compositional analysis, this can be costly, and is therefore, not readily accessible to every vineyard or winery [50,69]. These methods also rely on existing smoke taint marker compounds, but there is some doubt as to whether all of the compounds responsible for smoke taint have actually been identified [33,34,47]. Confounding the quantification of smoke taint, is the occurrence of some smoke taint marker compounds as natural constituents of grapes (to varying degrees, depending on grape variety [53,54]), and of oak wood, and therefore wines matured in oak barrels [36,37]. Thus, interpretation of data from smoke taint analyses depends on an understanding of both baseline volatile phenol levels present in fruit from different cultivars, as well as the potential contribution of volatile phenols from addition of oak chips or barrel aging. The Australian Wine Research Institute has been working on a “traffic light” system which aims to quantify the level of taint based on variety-specific baseline compound numbers generated from samples sourced across Australia. A limitation of this method is that baseline data is only available for smoke taint markers for ten cultivars [65]. Furthermore, these baselines are only representative of samples from Australia, and do not take into consideration the possible influence of terroir [70,71], so it is not yet clear if baselines are valid for all regions. It is prudent, therefore, for future research not to rely wholly on past results, and the existing suite of smoke taint marker compounds, but to continue to investigate the matrix from new angles and to gather data from a wider range of wine-producing regions and countries.

An alternative analytical approach which shows great promise for the rapid detection of smoke exposure is the use of remote sensing in the vineyard. A few contemporary research articles have recently been published describing rapid methods for monitoring smoke exposure in the vineyard. In 2019, Fuentes et al. investigated infrared thermography of vine canopies paired with near-infrared spectroscopy (NIRS) analysis of whole grapes and wine, to detect and quantify smoke taint [50]. The authors argued that these systems could be combined with machine learning to develop maps that could allow grape growers and winemakers to make informed decisions regarding harvest, and possibly even to sort fruit on the basis of the severity of smoke taint prior to processing. Mid-infrared spectroscopy (MIRS) has also been evaluated as a novel approach for detecting smoke taint in wine [72,73]. While classification rates of 61 and 70% were achieved for control and smoke-tainted wines, respectively, the ability of MIRS to discriminate wines was influenced by the level of smoke taint, as well as by grape variety and any oak maturation of wines [73]. Spectral methods offer considerably quicker and cheaper diagnostics than the traditional analytical methods that are currently available, although there are still some limitations with remote sensing. Firstly, NIRS analysis, like GC-MS and HPLC, relies on the use of specific smoke taint compounds as markers, i.e., compounds for which there is some doubt. Additionally, the system described in the Fuentes study was established for only seven cultivars, although the authors noted that this could be used for other cultivars with more data. Finally, the authors acknowledge that more research was needed before these systems could be used commercially [74]. A more recent study deployed commercial sensors in the vineyards for monitoring smoke exposure based on particulate matter concentrations [29]. Although these sensors did not accurately quantify the levels of smoke exposure, they gave an indication of the duration of smoke exposure, which would enable winemakers to make informed decisions about whether or not they should invest in more costly compositional analysis of grapes, where smoke exposure is found to have occurred, i.e., to determine the level of taint [29]. Another recent study investigated a method of remote drone sensing to assess smoke damage of vine canopies [75], but perhaps, in the future, this drone sensing could be adapted for assessment (and eventually quantification) of smoke exposure of grapes as well.

More recently, Fuentes et al. used an e-nose instrument, in combination with artificial intelligence, as a tool for the rapid assessment of smoke contamination of grapes and wine [76]. This approach could provide winemakers with timely information that could be used to implement amelioration strategies, thereby minimizing smoke taint in finished wines. While remote sensing technology has only been applied in preliminary studies to identify and to quantify smoke taint, to date, there is sufficient evidence to suggest these technologies warrant further investigation in the future.

With numerous studies predicting climate change will increase the duration and severity of future wildfires, not only in regions that have previously been impacted but new regions also, it is clear that smoke taint remains a significant challenge for the global wine industry [9,10,14,15,18,19]. Therefore, it is important to assess the sustainability of continuing to produce wines in fire-prone regions, and the reliance on preventative viticultural practices and ameliorative winemaking techniques. Consideration should also be given to the increasing risk of smoke taint in regions that may be more impacted in the future, in particular, Spain, Italy, and Portugal [16]. Managing the sustainability of wine production in fire-prone regions will depend on access to accurate climatic data for these regions, as well as a deeper understanding of both smoke taint and the methods available for amelioration of smoke taint [77]. A study conducted by Ponti et al., in 2018, used environmental and disease pressure data to analyze grape production within the context of climate change and provided an example of how modeling might be used to develop strategies to combat climate change within the agricultural sector [78]. Another study compared the sustainability of two Portuguese wines (one a terroir-focused wine, the other a wine produced on a massive scale) and contrasted data such as water usage and CO_2_ assimilation [79]. This model of analysis could be used to examine the sustainability of wines in regions susceptible to smoke exposure by compiling important fire-related data, such as rainfall, temperature, wind, and humidity, as well as the costs of implementing smoke taint remediation techniques. These analyses could provide vital insight into which wine regions (either impacted by, or vulnerable to, the effects of wildfires) will still be sustainable (both economically and environmentally) for wine production, and which areas will be unviable. It could also prompt an exploration into the investigation of new and unexploited grape-growing regions.

Research into smoke taint is still relatively young and significant knowledge gaps remain (outlined previously), which complicates both the quantification of smoke taint and evaluation of the remediation strategies that are currently available (namely, activated carbon fining and reverse osmosis). The lack of a ”silver-bullet” solution for smoke taint, coupled with forecasts of longer, more severe fire seasons, mean many prominent wine-producing regions around the world are at continued risk from the effects of fire and smoke. However, in the meantime, there are key lessons that have been learned and discoveries that have been made, primarily from experts based in Australia, Canada, and the United States, which can be applied in other countries if and when they are faced with the issue of smoke taint.

An important undertaking for wine regions around the world, but especially those that have not yet been impacted by fires and smoke taint, is compilation of baseline data for smoke taint marker compounds in fruit from different cultivars, particularly economically important grape varieties. Baseline data is not interchangeable between varieties and volatile phenol profiles are strongly influenced by the use of oak [29,58,80]. Further work is also needed to identify additional smoke volatiles that might be responsible for smoke taint, to ensure the optimal markers are being measured to quantify smoke taint, and more importantly, removed via remediation. Further investigation is also needed into the viability of remote sensing methods.

Other critical points for the protection of grape growers and winemakers which have not yet been directly reviewed, but that are inextricably linked to the issue of smoke taint, are the effectiveness of fire management strategies and the availability, value, and coverage of insurance policies. In areas where prescribed burns are routinely conducted as a method of fire prevention, it is important that there is communication between relevant stakeholders to ensure burns are not implemented near vineyards during the phenological stages at which vines are susceptible to the uptake of smoke. Furthermore, in the absence of an effective solution that guarantees the quality of wine made from grapes exposed to varying levels of smoke, in some instances winemakers have no option but to forego a vintage, in which case they incur significant financial losses. Thus, moving forward, it will also be important for smoke taint protection to be incorporated into insurance policies, and for improved lines of communication to be established between grape growers, winemakers, and fire management departments/agencies [19,22,23,81].

## 5. Conclusions

Despite the knowledge and technology that is currently available, there is no perfect solution for maintaining the quality of wine produced from smoke-affected grapes. Among the commercially available methods of remediation, activated carbon fining and reverse osmosis still appear to be the best options for amelioration of smoke-tainted wines, although the success of these methods has, thus far, been restricted to grapes and/or wines which exhibit low to moderate levels of smoke taint. For grapes that have been subjected to marginal smoke exposure and/or exposure at a low-risk stage of the growing cycle (pre-veraison, for example), cold maceration or limiting the duration of skin contact, together with careful yeast selection and/or aging with oak may enhance desirable organoleptic characteristics, and therefore yield a wine of acceptable quality. These approaches may, however, limit the style of wine that can be made, and therefore the economic returns (e.g., production of rosé wines rather than red wines, with less aging potential). Agricultural sprays such as biofilm [57] have shown promising results as vineyard-based preventative measures, but further research is needed to determine whether these sprays can be applied far enough in advance so as not to compromise the health and safety of vineyard workers during a fire. For more severely tainted wines, it is difficult, if not impossible, to produce quality wine with the methods described in this paper. Prevention of severely smoke-tainted grapes (and wine) might depend on external policies, such as improved forestry and fire management; alternatively, grape and wine producers might need to consider investing in crop insurance where coverage for smoke taint is available [81]. Ideally, these policies (and their respective policy makers) would work in conjunction with grape growers and winemakers to avoid smoke taint arising from prescribed burns, to reduce the incidence of wildfires, and/or to provide greater financial security for producers dealing with smoke taint. Future research should include elucidating the pathway by which smoke taint compounds enter grapes, as well as the specific compounds that are responsible for the sensory perception of smoke taint. These insights would aid the development of more precise methods of detection, prevention, and amelioration of smoke taint. Improved analytical methods, including remote sensing would enable winemakers to make informed decisions about whether or not to harvest grapes and/or how to manage smoke-affected fruit to achieve a saleable product. Alternative uses for smoke-tainted grapes, including the production of spirits via distillation or biofuels, could offer a pathway for grapes that cannot be used for winemaking. In response to the global Covid-19 pandemic, many distilleries produced alcohol-based sanitizers. This provides an alternate revenue stream for producers (albeit at a reduced income) when wine cannot be produced (or consumers cannot afford to buy them), and therefore a possible solution for smoke-tainted grapes.

## Data Availability

Not applicable.
